# Adherence to an Anti-Inflammatory Diet and Atopic Diseases’ Prevalence in Adolescence: The Greek Global Asthma Network Study

**DOI:** 10.3390/nu15143191

**Published:** 2023-07-19

**Authors:** Dafni Moriki, George Antonogeorgos, Despoina Koumpagioti, Despoina Chaloutsi, Philippa Ellwood, Luis García-Marcos, Kostas N. Priftis, Konstantinos Douros, Demosthenes Panagiotakos

**Affiliations:** 1 Allergology and Pulmonology Unit, 3rd Pediatric Department, National and Kapodistrian University of Athens, 12462 Athens, Greece; dmoriki@med.uoa.gr (D.M.); geanton@hua.gr (G.A.); ds215068@hua.gr (D.C.); kpriftis@med.uoa.gr (K.N.P.); 2 Department of Nutrition and Dietetics, School of Health Sciences and Education, Harokopio University, 17676 Athens, Greece; dbpanag@hua.gr; 3 Department of Nursing, National and Kapodistrian University of Athens, 11527 Athens, Greece; dkoumpagioti@nurs.uoa.gr; 4 Department of Pediatrics: Child and Youth Health, Faculty of Medical and Health Sciences, University of Auckland, Auckland 1023, New Zealand; p.ellwood@auckland.ac.nz; 5 Pediatric Allergy and Pulmonology Units, ‘Virgen de la Arrixaca’ University Children’s Hospital, University of Murcia, 30100 Murcia, Spain; lgmarcos@um.es; 6 Biomedical Research Institute of Murcia (IMIB-Arrixaca), 30120 Murcia, Spain; 7 Network of Asthma and Adverse and Allergic Reactions (ARADyAL), 28029 Madrid, Spain

**Keywords:** diet, Mediterranean, lifestyle, atopic, asthma, allergies

## Abstract

Objective: Atopic diseases are among the most common morbidities in children and adolescents. The association between adherence to an anti-inflammatory dietary pattern and the prevalence of atopic diseases among adolescents was examined. Methods: A total of 1934 adolescents (boys: 47.5%, mean age (standard deviation): 12.7 (0.6) years) were voluntarily enrolled. Participants completed a validated questionnaire on atopic disease status as well as one assessing dietary habits and other sociodemographic and lifestyle characteristics. A special Diet Anti-inflammatory Index (DAI) score was calculated for the evaluation of adherence to an anti-inflammatory dietary pattern. Results: A total of 6.9% of the participants reported current asthma symptoms, while 25.3% reported rhinitis symptoms and 8.9% reported eczema. Adolescents with high adherence to an anti-inflammatory diet were 58% less likely to have asthma symptoms compared with those with low adherence when adjusted for multiple confounders (*p* < 0.01). No significant associations were observed between the level of adherence to an anti-inflammatory diet and the prevalence of allergic rhinitis and eczema. Conclusion: An anti-inflammatory diet seems to be independently associated with a lower prevalence of asthma in adolescents. Thus, pediatricians and other healthcare providers should promote anti-inflammatory dietary patterns as a preventive measure for atopic diseases from early stages of life.

## 1. Introduction

The prevalence of atopic diseases such as asthma, allergic rhinitis, and eczema has increased among children and adolescents in the past decades [1,2]. This trend is generally attributed to environmental and lifestyle changes that render children susceptible to immune-mediated disorders [3]. Diet has emerged as a key factor in human health and has been associated with various chronic diseases, including atopic diseases [4]. In particular, evidence has accumulated to support the idea that certain nutrients and food components, such as fatty acids, vitamins, and fiber, can modulate the immune system and thus may enhance or prevent the development of atopic diseases [5].

So far, specific food groups and dietary patterns have been inversely associated with atopic diseases in both children and adolescents. High fruit and vegetable consumption, as well as adherence to healthy dietary patterns such as the Mediterranean diet, have been shown to have a protective effect against asthma [6,7] and allergic rhinitis [7,8]. Conversely, the long-term consumption of an unhealthy diet that is characterized by low intake of fruits and vegetables as well as high consumption of refined grains and saturated fat has been associated with an increased risk of asthma in preschool children [9].

A growing body of evidence suggests that diet influences oxidative stress and chronic inflammation, which are considered determinants of risk and mortality of most non-communicable diseases [10]. The Dietary Inflammatory Index (DII) has been proposed as a tool to measure the inflammatory potential of a diet [11]. This scoring system was developed to consider an individual’s diet as a whole rather than specific foods or nutrients, thus better assessing the effect of a diet on specific health outcomes. The association of DII with the prevalence and severity of atopic diseases in adults is well documented, especially asthma. In particular, an elevated DII score, which is indicative of a pro-inflammatory diet, has previously been associated with an increased risk of asthma and asthma symptoms [12,13], reduced lung function [14], and increased all-cause mortality in adult asthmatic patients [15]; however, no data exist in pediatric and adolescent populations.

The aim of this study was to investigate the association between the level of adherence to an anti-inflammatory dietary pattern and the prevalence of asthma, allergic rhinitis, and eczema in Greek adolescents in the context of the Global Asthma Network (GAN) study [16].

## 2. Materials and Methods

### 2.1. Design

The GAN study is a population-based, epidemiological study aimed at evaluating the global prevalence, severity, management, and risk factors of asthma and other atopic diseases, such as allergic rhinitis and eczema, in two specific age groups, i.e., 6–7 and 13–14 years [16]. The Greek cohort of the GAN study followed the same protocol as the GAN Phase I study but only included adolescents aged 13–14 years [17].

### 2.2. Setting and Sampling

The sampling took place in the high school setting from February to March 2020 in the Athens greater metropolitan area. A two-stage convenience sampling scheme was applied. First, twenty high schools were randomly chosen from a list of schools that was provided by the Secondary Education Office of the Ministry of Education (5% of all schools). Then, all first and second grade students (a total of 2560 adolescents) at each high school were invited to participate and provide informed consent from their parents or guardians. Special schools for children with disabilities or special educational needs were not included because no permission was granted by the Secondary Education Office.

### 2.3. Sample

A total of 2560 adolescents were invited to participate in the study; a total of 1934 adolescents (921 boys, 47.5%; 1013 girls, 52.5%) with a mean age of 12.7 (0.6) (standard deviation, (SD)) years participated in the study (participation rate of 76%).

### 2.4. Measurements

All participants were clinically examined in the school setting by trained pediatricians and nurses (i.e., the study’s investigators). Following the clinical examination, all participants completed the standardized and validated GAN study questionnaire for adolescents (i.e., 13–14 years old) [18]. The core questions for the assessment of asthma, rhinitis, and eczema symptoms were formed according to the International Study of Asthma and Allergies in Childhood (ISAAC) instructions; more information on the questionnaire validation is available on the GAN website [19]. Current asthma was defined as a positive answer to the question: “Have you had wheezing or whistling in the chest in the past 12 months?”. Similarly, current rhinitis was defined as a positive answer to the question: “In the past 12 months, have you had a problem with sneezing or a runny or blocked nose when you did not have a cold or the flu?”. Current eczema was defined as a positive answer to the question: “Have you had this itchy rash at any time in the past 12 months?”. The rationale for a 12-month reference period was to reduce recall errors. Furthermore, this period was considered to be independent of the month in which the questionnaire was completed [19].

The GAN study evaluation also contained questions about the participants’ dietary habits and physical activity status, as well as information about their living environment and number of siblings. Specifically, the consumption frequency of various food groups or food items that are usually consumed in Greece (i.e., meat and products; fish and other seafood; fruits; cooked vegetables (green and root); raw vegetables and salads (green and raw); pulses (beans, peas, lentils); dairy; cereals; candies, lollies, and sweets; and fizzy or soft drinks) during the previous 12 months was recorded using the study’s validated food frequency questionnaire (FFQ) [19]. Adherence to an anti-inflammatory dietary pattern was evaluated using a special diet score that was developed according to the DII methodology [11], and calculated for each participant based on the information provided through the FFQ. In particular, all major food items, food groups (i.e., cereals, fish and seafood, fruits, cooked and raw vegetables, pulses, nuts, milk and dairy products, olive oil), and beverages (juices, soft drinks), totaling 14 items, were included in the FFQ, and they were assigned scores of −1, +1, and 0 depending on whether they had a pro-inflammatory effect, anti-inflammatory effect, or no effect, respectively, according to food composition tables [11]. Following this procedure, a specific z-score was calculated for each food or beverage according to the participants’ consumption and then multiplied by the previously indicated score values. The sum of these products was used to create the Diet Anti-inflammatory Index (DAI) score; then, the score was transformed to a range spanning 0 to 100. This approach for the development of a diet anti-inflammatory or inflammatory index using the information provided from a FFQ has been applied before and has been tested for its validity [20]. For interpretation reasons, three groups of DAI scores were used to classify adolescents as having “low” (score < 33), “moderate” (score 34–67), or “high” (score > 67) adherence to an anti-inflammatory diet.

Physical activity status was evaluated using questions that assessed the number of times per week that the participants engaged in vigorous physical activities (i.e., to the point of feeling shortness of breath); the hours per day that they watched television, movies, or videos; and the number of hours per day that they engaged in computer and Internet activities (gaming, chatting, Facebook, YouTube, etc.). Adolescents were then categorized as having an active or sedentary lifestyle according to the GAN study protocol [17].

Height and weight were measured by the study’s investigators while following standard procedures, and body mass index (BMI) was determined in order to categorize participants as normal weight, overweight, and obese; the International Obesity Task Force (IOTF) classification was applied [21].

### 2.5. Power Analysis

The number of enrolled adolescents was sufficient to achieve a statistical power of 92% for assessing one standardized difference in the DAI between participants with an atopic disease and the remaining participants at a probability level of <0.05 (two-sided hypotheses).

### 2.6. Statistical Analysis

SPSS 28 software was used to perform all statistical analyses (IBM SPSS Statistics, version 28.0.1.1). Normally distributed variables are presented as the mean values (and standard deviation, SD), and categorical variables are presented using absolute (and relative) frequencies. The Pearson’s chi-square and Fisher’s exact tests were applied when they were appropriate to examine the associations among atopic outcomes, the anthropometric and lifestyle characteristics of the adolescents, and the level of adherence to an anti-inflammatory diet (low, moderate, and high). A logistic regression analysis was performed to evaluate the association between the level of adherence to an anti-inflammatory diet (DAI score) and the atopic outcomes. The results are expressed as odds ratios (OR) with 95% confidence intervals (CIs). The residuals were calculated to assess the model’s goodness-of-fit.

A hierarchical discriminant analysis was performed to further investigate the classification ability of DAI components in characterizing participants according to atopic disease outcomes. The analysis yielded a discriminant function (i.e., Fisher’s discriminant function) as a linear combination of the predictor variables that provide the best discrimination for the outcome variable. Wilk’s lambda was calculated to determine which DAI components had better classification ability (the lambda ranges from 0 to 1; values that are closer to 0 indicate that the examined factor has good classification ability, while values that are closer to 1 indicate that the factor has poor ability to classify cases in each group). All reported probability values (*p*-values) were based on two-sided hypotheses and were compared at a significance level of 5%.

## 3. Results

### 3.1. Prevalence of Atopic Diseases

Of the 1934 adolescents who participated in the study, 6.9% reported current asthma symptoms (at least one episode of wheezing or whistling in the chest in the past 12 months), 25.3% reported current rhinitis symptoms (at least one episode of sneezing or runny nose without cold symptoms in the past 12 months), and 8.9% reported current eczema symptoms (the appearance of an itchy rash in at least the past 12 months).

### 3.2. Anti-Inflammatory Diet and Atopic Diseases

The mean (SD) DAI score was 73.7 (18.7) for boys and 72.6 (19.8) for girls (*p* = 0.559), which may be considered moderate to high (i.e., 73.7/100). Half of the adolescents were classified as having “high” adherence (i.e., 50%), with nearly a third of them having “moderate” adherence (i.e., 33.7%) and only 16.3% having “low” adherence to an anti-inflammatory dietary pattern. Table 1 shows the prevalence of atopic diseases, as well as various demographic, and lifestyle characteristics, according to the level of adherence to an anti-inflammatory diet. Significantly higher prevalence rates of current asthma were observed in adolescents with low adherence to an anti-inflammatory diet compared with those with high adherence. In addition, adolescents with high adherence tended to be more physically active compared with those with low adherence; however, the difference was not significant (*p* > 0.05).

Due to the observational design of the study, residual confounding errors may exist. Thus, in Table 2, the results from the nested, multi-adjusted logistic regression models are presented, which evaluated the association between the level of adherence to an anti-inflammatory diet and the prevalence of current asthma, allergic rhinitis, and rash symptoms as outcomes. Compared with low adherence, adolescents with high adherence to an anti-inflammatory diet had 58% lower odds of having any asthma symptoms when adjusted for several confounders (Model 3, Table 2). No significant interactions were observed between sex, obesity, the physical activity status of adolescents, parental education status, and DAI score on asthma prevalence, suggesting that no moderating effect exists. No significant associations were observed between current allergic rhinitis and current eczema with respect to any level of adherence to an anti-inflammatory diet.

Regarding the other factors included in the models, parental educational level was associated with a lower likelihood of allergic rhinitis symptoms, while an active physical activity lifestyle was associated with a lower likelihood of allergic rash symptoms. In contrast, the presence of an older sibling and indoor exposure to dampness and/or mold were associated with a higher likelihood of presenting allergic rash symptoms (Table 2).

### 3.3. Decomposition of an Anti-Inflammatory Diet in Relation to Atopic Diseases

The hierarchical discriminant analysis was used to determine which of the DAI components were most strongly associated with classifying adolescents as having an atopic disease. Based on the Wilk’s lambda values obtained from the discriminant analysis, it was revealed that the consumption of pulses (λ = 0.502), followed by raw vegetables (λ = 0.358), milk (λ = 0.307), and fruit (λ = 0.211) consumption, were the food groups with the highest discrimination ability among those that were studied.

## 4. Discussion

The hypothesis that adherence to an anti-inflammatory diet is associated with a reduced prevalence of asthma, allergic rhinitis, and eczema was evaluated in adolescents aged 13–14 years in the context of the Greek part of the GAN study. High adherence to an anti-inflammatory dietary pattern, as reflected by a high DAI score, was strongly associated with reduced asthma prevalence. The latter association persisted even after adjusting for potential confounders, including obesity status and physical activity level. Conversely, no significant associations were recorded for allergic rhinitis and eczema, which is probably related to the lack of systemic inflammation in mild eczema and allergic rhinitis [22] as well as the governing influence of other non-dietary factors, such as genetic predisposition, which have been shown to modify the effect of a diet on atopic diseases [23]. Asthma is the most common chronic disease in children, having an estimated prevalence of approximately 10% worldwide, and it imposes a significant burden on children and their families [24]. The finding that adolescents with high adherence to an anti-inflammatory diet were 58% less likely to have current asthma compared with those with low adherence is of substantial importance, as a considerable proportion of adolescents could probably have been protected by adhering to a healthy dietary pattern. Therefore, despite the potential limitations of our study, the data presented have important public health implications as they suggest a powerful preventive measure for asthma in adolescence.

Over the past few decades, extensive research has been conducted on the role of the Mediterranean diet on human health and longevity. The protective effects of this traditional dietary pattern in atopic diseases, especially in asthma, are already known [6]. The main aspects of this dietary pattern include a relatively high consumption of fruits and vegetables, whole-grain cereals, pulses, nuts, fish, and olive oil, which are known to have antioxidant and anti-inflammatory properties [25]. However, the Mediterranean diet is not a sustainable dietary pattern in other parts of the world as it cannot be widely accepted and adopted; it is, however, sustainable for the populations of Mediterranean countries, where the mild climate favors the production of the Mediterranean foods throughout the year. However, other similar dietary choices should be promoted to achieve the health benefits that this dietary pattern has. Over the past few years, growing evidence has suggested that diet is one of the most important regulators of chronic inflammation that is associated with the development of various diseases. Therefore, Shivappa and colleagues suggested that in order to compare the diets of different populations, it is better to assess their inflammatory potential rather than trying to classify them into specific dietary patterns that are difficult to universally adopt [11]. Studies evaluating the effects of an anti-inflammatory diet on the prevalence and severity of allergic diseases in children are very sparse in the literature. In particular, a pro-inflammatory diet, as reflected by a high DII score, has been previously associated with an increased risk of atopic dermatitis [26], allergic rhinitis [23], and atopic wheeze in children [13]. Moreover, in a recent cross-sectional study of children aged 5–14 years, a pro-inflammatory diet was associated with a high burden of asthma symptoms [27]. Our findings support a protective association between the level of adherence to an anti-inflammatory diet and asthma among adolescents living in a Mediterranean country, one that is independent of other potential lifestyle confounding factors. The fact that background dietary habits of participating adolescents were close to a Mediterranean type of diet enhances the findings of the present analysis towards the protective association of an anti-inflammatory dietary pattern, which is not necessarily due to it being Mediterranean-type. Moreover, foods with high anti-inflammatory content can be found in all parts of the world; therefore, the results promote their consumption. The discriminant analysis applied in this work revealed that food choices such as the consumption of pulses, raw vegetables, milk, and fruits support the previous statement about the sustainability of an anti-inflammatory diet, as they are the groups with a higher impact on asthma prevalence.

Atopic diseases such as asthma, allergic rhinitis, and eczema are characterized by a specific pattern of inflammation that is mainly mediated by immunoglobulin E (IgE)-dependent pathways. IgE is released by B lymphocytes (B-cells) in the presence of several interleukins (ILs) and cytokines that are produced by T-helper lymphocytes. IL-4 and IL-13 produced by type 2 T-helper cells (Th2) induce the class-switching of B-cells to IgE production, whereas IFN-γ produced from type 1 T-helper (Th1) cells inhibits this switching. Other mediators, such as IL-5 (which promotes eosinophilic inflammation) and IL-9 (which stimulates the proliferation of mast cells), are also involved [28]. Regulatory T-cells (Tregs) are a subset of T-cells that suppress immune responses, thereby contributing to the maintenance of homeostasis and self-tolerance. In allergic patients, a defect in Tregs has been observed, which may further promote Th2 cell activation [29]. Anti-inflammatory dietary patterns are rich in nutrients such as unsaturated fatty acids, e.g., mono- and *n*-3 polyunsaturated fatty acids (PUFAs), antioxidants, carbohydrates, and fiber, which could prevent allergy by modulating the inflammatory process. The anti-inflammatory effect of *n*-3 polyunsaturated fatty acids is thought to be exerted through the reduction of prostaglandin 2 (PGE2) levels, which may subsequently reduce Th2-related cytokines and IgE levels [30]. Antioxidants are chemical compounds that inhibit free radicals and can modulate the immune and inflammatory pathways that are associated with atopic diseases; examples of antioxidants include vitamins (i.e., A, C, E), selenium, β-carotene, and flavonoids, which are abundant in fruits and vegetables. Vitamin C exerts its antioxidant effect by inactivating oxygen free radicals and by inhibiting the secretion of superoxide anions by macrophages [31]. Vitamin E and selenium have been shown to affect Th2 cytokines (IL-4, IL-5, and IL-13) as well as their upstream cytokines (IL-25 and IL-33), suggesting that they may be a beneficial treatment for the management of allergic diseases such as allergic rhinitis and asthma [32]. In addition, fruits and vegetables are rich in fiber, which is fermented by gut bacteria to produce metabolites such as short-chain fatty acids (SCFAs). These metabolites have immunomodulatory properties, including their ability to activate G-protein-coupled receptors (GPCRs) that have been shown to affect airway inflammation and airway reactivity in animal models [33] and their ability to induce Tregs that are necessary to suppress Th2 immune responses [34]; however, the complex interactions between nutrients and the immune system remain largely unknown, and this scientific field is open for further research.

A major limitation of the present study is that adherence to an anti-inflammatory diet was assessed through the DAI score, which is a shorter version of the original DII methodology. Moreover, the GAN study has a cross-sectional design and therefore suffers from the inherent limitations that occurs from this type of observational study, such as non-response, recall bias, and the inability to make causal inferences. However, an effort was made to not overinterpret the results. Reverse causality bias may also occur, as adolescents with atopic diseases may have adopted healthier eating habits as recommended by their physicians. Finally, although all known cofounders were included in the analyses, residual confounding phenomena cannot be excluded.

## 5. Conclusions

Our study suggests a protective association between the level of adherence to an anti-inflammatory diet and the prevalence of asthma symptoms in adolescents. As dietary habits are a modifiable risk factor, particularly for the age groups studied in this work, healthcare providers and pediatricians in particular should encourage and promote the adoption of anti-inflammatory dietary patterns from the very early stages of life as an important preventive measure in the management of atopic diseases.

## Figures and Tables

**Table 1:** Distribution of anthropometric, lifestyle, and atopic characteristics by the level of adherence to an anti-inflammatory diet among adolescents aged 13–14 years (n = 1934).

	Adherence to an Anti-Inflammatory Diet (Based on the DAI Score)			
	Low	Moderate	High	*p*-Value
N	315	652	967	
Boys, % *	45.4	49.4	47.2	0.465
**Atopic diseases prevalence**				
Asthma symptoms in the past 12 months (current)	10.8	8.3	4.6	<0.001
Allergic rhinitis symptoms in the past 12 months (current) Allergic rash symptoms in the past 12 months (current)	29.2 8.9	24.7 9.5	24.5 8.5	0.225 0.809
Overweight/Obese adolescents, % yes	33.0	31.6	32.6	0.881
Adherence to an active physical activity lifestyle, % yes **	19.4	21.6	25.2	0.056
**Family characteristics**				
Having an older sibling, % yes	42.9	41.0	44.2	0.458
Parental atopic history, % yes	8.9	8.1	7.6	0.786
Parents ever smoking, % yes	54.9	54.8	57.0	0.636
Parental educational level, % tertiary	65.6	66.0	66.6	0.936
Indoor exposure to dampness and/or mold, %yes	21.9	24.5	23.8	0.667

* Results are presented as the mean ± SD and relative frequencies. Mean values were compared using an ANOVA test, and associations between categorical variables were evaluated using the chi-square test. ** Participating in vigorous physical activity for >3 h/day plus watching TV and engaging in computer and Internet activities for <3 h.

**Table 2:** Results from the multi-adjusted logistic regression models (OR, 95% CI) assessing the association between a history of atopic disease (asthma, rhinitis, eczema) in the previous 12 months and adherence to an anti-inflammatory diet in adolescents (n = 1934).

	Asthma Symptoms in the Past 12 Months (Current Asthma)			Allergic Rhinitis Symptoms in the Past 12 Months (Current Allergic Rhinitis)			Allergic Rash Symptoms in the Past 12 Months (Current Eczema)		
	Model 1	Model 2	Model 3	Model 1	Model 2	Model 3	Model 1	Model 2	Model 3
Adherence to DAI									
Moderate vs. Low	0.75 (0.48–1.17)	0.76 (0.48–1.21)	0.77 (0.49–1.22)	0.79 (0.59–1.07)	0.79 (0.59–1.08)	0.79 (0.59–1.08)	1.08 (0.68–1.72)	1.07 (0.67–1.72)	1.09 (0.68–1.75)
High vs. Low	0.41 * **(0.26–0.65)**	**0.42** **(0.26–0.67)**	**0.42** **(0.26–0.68)**	0.79 (0.60–1.05)	0.80 (0.60–1.07)	0.80 (0.60–1.06)	0.97 (0.62–1.51)	0.93 (0.59–1.46)	0.96 (0.61–1.51)
Boys vs. Girls		0.96 (0.67–1.37)	0.99 (0.69–1.41)		0.90 (0.74–1.11)	0.92 (0.75–1.13)		1.33 (0.96–1.83)	1.32 (0.96–1.83)
Having an older sibling (Yes vs. No)		1.01 (0.71–1.45)	0.99 (0.69–1.43)		0.88 (0.71–1.09)	0.89 (0.72–1.09)		**1.45** **(1.05–1.99)**	**1.42** **(1.03–1.95**)
Atopic history of parents (Yes vs. No)		0.70 (0.33–1.46)	0.69 (0.33–1.45)		1.10 (0.76–1.60)	1.11 (0.77–1.61)		1.12 (0.64–1.96)	1.10 (0.63–1.93)
Smoking (ever) of parents (Yes vs. No)		0.97 (0.68–1.38)	0.97 (0.68–1.39)		0.95 (0.77–1.16)	0.95 (0.77–1.17)		0.93 (0.68–1.28)	0.93 (0.68–1.28)
Educational level of parents (Tertiary vs. Lower)		0.63 (0.44–0.91)	0.69 (0.48–1.00)		**0.78** **(0.63–0.97)**	**0.77** **(0.62–0.96**)		1.19 (0.85–1.68)	1.31 (0.92–1.85)
Indoor exposure to dampness and/or mold (Yes vs. No)		1.26 (0.84–1.88)	1.27 (0.85–1.89)		1.23 (0.97–1.56)	1.24 (0.97–1.57)		**1.66** **(1.19–2.33)**	**1.67** **(1.19–2.35)**
Overweight/Obesity vs. Normal weight			1.41 (0.97–2.03)			1.14 (0.91–1.42)			1.04 (0.74–1.46)
Physically active lifestyle ** (Yes vs. No)			0.67 (0.40–1.10)			1.16 (0.90–1.48)			**0.56** **(0.36–0.87)**

Model 1: Age-adjusted. * Numbers in bold indicate *p* < 0.05. ** Participating in vigorous physical activity for >3 h/day plus watching TV and engaging in computer and Internet activities for <3 h per day (n, %).

## Data Availability

The datasets generated and/or analyzed during the current study are part of the Global Asthma Network study and will be made available on the GAN website within 12 months of the publication of all GAN Phase I analyses. Access will require a formal request through the website, a written proposal, and a signed data access agreement.
